# Better stoma care using the Stoma App: does it help? A first randomized double-blind clinical trial on the effect of mobile healthcare on quality of life in stoma patients

**DOI:** 10.1007/s00464-023-10593-x

**Published:** 2024-01-08

**Authors:** Sebastiaan L. van der Storm, Esther C. J. Consten, Marc J. P. M. Govaert, Jurriaan B. Tuynman, Steven J. Oosterling, Brechtje A. Grotenhuis, Anke B. Smits, Hendrik A. Marsman, Charles C. van Rossem, Eino B. van Duyn, Lindsey C. F. de Nes, Emiel Verdaasdonk, Tammo S. de Vries Reilingh, Wouter Vening, Willem A. Bemelman, Marlies P. Schijven, the Stoma APPtimize collaborative study group, Liesbeth W. E. Boerman, Noor E. van den Broek, Ivonne J. C. M. Botman, Danielle Verhoeven, Valeria Baars, Claudia van Tienderen, Patricia van Bottenberg, Judith Hartog, Christianne J. Buskens, Roel Hompes, Miranda Kusters, Marieke S. Walma, Bono Meijs

**Affiliations:** 1 Surgery, Amsterdam UMC, Location University of Amsterdam, Meibergdreef 9, Amsterdam, The Netherlands; 2 Amsterdam Gastroenterology Endocrinology Metabolism, Amsterdam, The Netherlands; 3 Amsterdam Public Health, Digital Health, Amsterdam, The Netherlands; 4 Surgery, Meander Medical Center, Amersfoort, The Netherlands; 5 Surgery, University Medical Center Groningen, Groningen, The Netherlands; 6 Surgery, Dijklander Ziekenhuis, Hoorn, The Netherlands; 7 Surgery, Amsterdam UMC - Location VUmc, Amsterdam, The Netherlands; 8 Surgery, Spaarne Gasthuis, Hoofddorp, The Netherlands; 9 Surgery, Antoni van Leeuwenhoek Ziekenhuis, Amsterdam, The Netherlands; 10 Surgery, Antonius Ziekenhuis, Nieuwengein, The Netherlands; 11 Surgery, OLVG Hospital, Amsterdam, The Netherlands; 12 Surgery, Maasstad Ziekenhuis, Rotterdam, The Netherlands; 13 Surgery, Medisch Spectrum Twente, Enschede, The Netherlands; 14 Surgery, Maasziekenhuis Pantein, Boxmeer, The Netherlands; 15 Surgery, Jeroen Bosch Ziekenhuis, ’s-Hertogenbosch, The Netherlands; 16 Surgery, Elkerliek Ziekenhuis, Helmond, The Netherlands; 17 Surgery, Rijnstate Ziekenhuis, Arnhem, The Netherlands; 18 Department of Surgery, Amsterdam Gastroenterology and Metabolism, Amsterdam Public Health, Digital Health, Amsterdam UMC, University of Amsterdam, Amsterdam, The Netherlands

**Keywords:** Stoma, Colorectal surgery, Ehealth, Mhealth, Mobile application, Quality of life

## Abstract

**Background:**

Receiving a stoma significantly impacts patients’ quality of life. Coping with this new situation can be difficult, which may result in a variety of physical and psychosocial problems. It is essential to provide adequate guidance to help patients cope with their stoma, as this positively influences self-efficacy in return. Higher self-efficacy reduces psychosocial problems increasing patient’s quality of life. This study investigates whether a new mobile application, the Stoma App, improves quality of life. And if personalized guidance, timed support, and peer contact offered as an in-app surplus makes a difference.

**Methods:**

A double-blind, randomized controlled trial was conducted between March 2021 and April 2023. Patients aged > 18 years undergoing ileostomy or colostomy surgery, in possession of a compatible smartphone were included. The intervention group received the full version of the app containing personalized and time guidance, peer support, and generic (non-personalized) stoma-related information. The control group received a restricted version with only generic information. Primary outcome was stoma quality of life. Secondary outcomes included psychological adaption, complications, re-admittance, reoperations, and length of hospital stay.

**Results:**

The intervention version of the app was used by 96 patients and the control version by 112 patients. After correction for confounding, the intervention group reported a significant 3.1-point improvement in stoma-related quality of life one month postoperatively (*p* = 0.038). On secondary outcomes, no significant improvements could be retrieved of the intervention group.

**Conclusion:**

The Stoma App improves the quality of life of stoma patients. Peer support and personalized guidance are of significant importance in building self-efficacy. It is to be recommended to implement Stoma app—freely available software qualifying as a medical device—in standard stoma care pathways for the benefits of both patients and healthcare providers.

In the Netherlands, it is estimated that over 7000 new stomas are created every year [1]. Ileostomies or colostomies may be necessary for patients with colorectal malignancy, inflammatory bowel disease, or to resolve or mitigate intestinal leakage for other reasons. Getting a stoma may negatively impact patients’ self-image and daily functioning, leading to a reduced quality of life [2–4]. In the initial postoperative period, patients must learn to cope and adapt to the new situation which can be challenging. This may result in several psychosocial problems such as insecurity, depression, stress, anxiety, decreased social participation, and sexual problems [5]. Patients are also at risk of stoma-related morbidity which has an incidence of 20–80%, the most common complications are peri-stomal skin problems and leakages [6, 7]. Complications themselves can exert a significant negative impact on the mental and social well-being of patients [8].

Self-efficacy has been found to be very important for patients having a stoma. When self-efficacy is high, psychosocial problems and stoma-related morbidities are effectively reduced [9, 10]. Therefore, it is crucial to provide adequate patient education and guidance, especially in the immediate preoperative and postoperative period. Several educational interventions have demonstrated positive results in terms of enhancing psychosocial skills, self-efficacy, and quality of life [11, 12]. However, providing adequate stoma care or obtaining information in the out-of-hospital setting can be challenging. In general, Dutch patients reported only moderate satisfaction with the stoma care they received, highlighting several shortcomings in information provision and postoperative care. Also, they express a need to be in contact with peer patients [13, 14].

A mobile application (app) may act as a medical device and has great potential to improve and support healthcare [15]. Introducing a personalized app as an addition to regular stoma care can provide stoma patients with important benefits. These benefits include easily accessible information that relates to specific circumstances, and the opportunity to engage in peer-to-peer contact with other patients in a safe, anonymous environment, if one should desire so [13, 14]. Providing reliable and understandable information on stoma management is very important for patients. This should include what is considered to be ‘normal’ and what is not, along with the possibility for patients to interact and learn from other patients in the same situation (peers). Having access to such information at any time may contribute to acceptance, self-confidence, and self-efficacy, enabling patients to regain control of their new situation. In turn, this may reduce the demand for caregivers and potentially avoid returning to the clinic.

The app ‘Stoma App’ offers a wide range of relevant stoma-related information. It provides personalized and timed guidance and facilitates peer-to-peer patient contact. It includes—among others—step-by-step videos on how to take care of a stoma, information on stoma materials, nutrition, exercise, emotional and sexual well-being, and traveling. One is also able to self-monitor progress in stoma self-care. The layout of the full version of the Stoma App is depicted in Fig. 1.

**Fig. 1:**
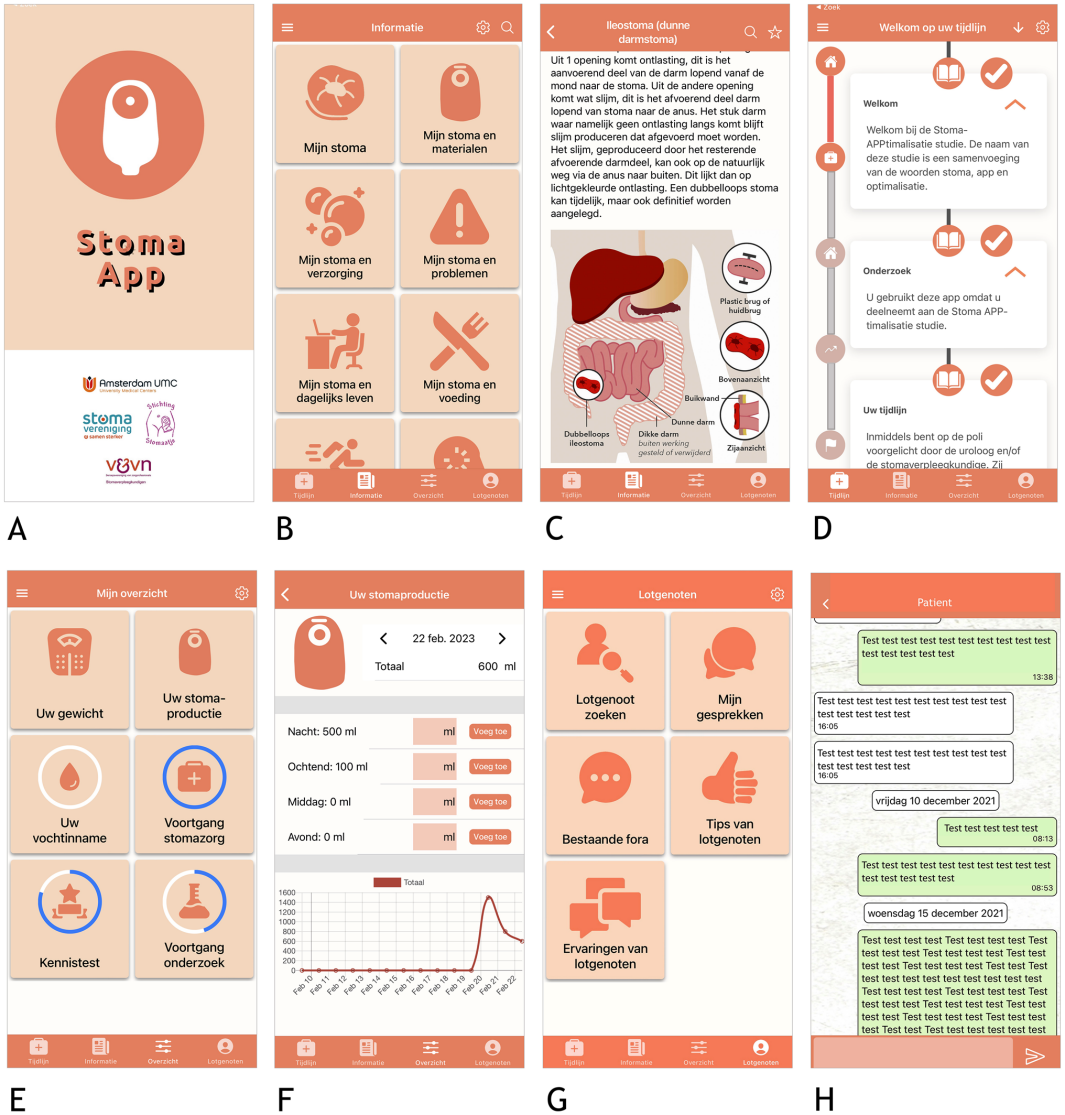
Screenshots of the Stoma App (in the Dutch language). **A** The splash screen when starting up the app shows the cooperating patient and professional associations. **B** The information library containing relevant information. **C** Information and illustration of an ileostomy. **D** The personalised information timeline which is personalised based on the type of stoma, operation setting, and operation indication, and timed based on the operation and hospital discharge dates read text boxes are ticked off and the left bar illustrates the patients process in the pathway (in this case, in admission). **E** “My overview” in which patients can enter their process. **F** Registration of the stoma production. **G** Peer-support platform, the app provides a suggestion list of peers which is based on the type of stoma, operation indication, age, and sex, all of which can be contacted **H**) One–one peer chat

The Stoma App is based on the Dutch Ostomy Care Guidelines and built with patients and providers and caters to various patients’ needs [12, 13]. By conducting this double-blind randomized controlled trial (RCT), we aimed to investigate whether personalized and timed guidance and peer contact in a patient-centered app significantly improve the Stoma quality of life (Stoma QoL).

## Methods

### Study setting

The Stoma APPtimize trial is a double-blind multicenter randomized controlled trial that was conducted since March 2021 in two academic hospital centers and across twelve teaching hospitals in the Netherlands. Data collection for the short-term outcomes was completed in April 2023. The study was approved by the local medical ethics committee of Amsterdam UMC registration number NL75119.018.20). The study protocol has been published previously [16]. The study is reported according CONSORT-EHEALTH (Consolidated Standards of Reporting Trials of Electronic and Mobile Health Applications and online TeleHealth) checklist [17].

### Study population

Patients were eligible if they received an elective or emergency ileostomy or colostomy, were aged 18 years or older, and had a smartphone operating on at least iOS 9 or Android 8.0. Patients who met one or more of the following criteria were not considered for inclusion:

Exclusion criteria:

- Patients with a Karnofsky performance score ≤ 40.
- Incompetence of understanding the Dutch language.
- Visual impairment, unless well corrected with visual aids.
- Physical disabilities limiting the use of a mobile app, such as Parkinson’s disease.
- Patients with pre-existing skin conditions, such as pemphigus, para-pemphigus, and psoriasis.

### Group allocation and blinding

After inclusion, participants were provided with a unique generated access code that blindly randomized them to either the intervention or the control (1:1) group using block sizes of two, four, and six. Randomization was stratified for indication for surgery (benign or malignant) and type of stoma (ileostomy or colostomy). Only the coordinating researcher was unblinded as he provided the app’s instructions. Participants were instructed not to tell other participants or patients about the content of their version of the app. Participants used the app according to their own preferences without any intervention of the research team; however, they had the option the contact the research team for technical support if needed.

### Procedures

Participants were supported by the app ‘Stoma App’ immediately after inclusion until three months postoperative or until stoma reversal. The intervention group had access to the full version of the app. In this version, information is provided in a generic information library and personalized timeline triggering push notifications. These push notifications were used to inform and activate patients at specific times. All information could be recalled at any moment in time. Participants could watch instruction videos on stoma care and register their weight, fluid intake, stoma production, and the process of stoma self-care. Participants also had the option to interact anonymously with other patients (who used the public, restricted version of the app).

The control group received a restricted version of the app that contained generic stoma-related information, lacking personalization and timing. This information was comparable with the standard patient information folders typically used in the Netherlands. Both groups were required to complete questionnaires through the app. The Stoma App is CE marked (NL-CA002-2020-53630), complies with the General Data Protection Regulation, and follows ISO 27001 data and security guidelines [18].

### Outcome

The primary outcome is quality of life, measured with the validated Stoma QoL questionnaire. To correct for potential cofounding on digital literacy, participants completed a questionnaire on their mobile proficiency [19]. Secondary outcome measures included psychological adaptation, postoperative outcomes, stoma-related problems, and number of contact moments with the ostomy nurse at the outpatient clinic. All study outcomes and their assessments are described in the study protocol [16].

### Statistical analysis

The sample size was calculated based on the Stoma QoL score of 56.6 as retrieved as baseline from a previous study and the hypothesis that the Stoma QoL of the Stoma APPtimize group would increase to 61.6 [20]. Using a sample size calculation with 90% power, a 2-sided alpha of 0.05, and a standard deviation of 10, 7, we estimated that 98 participants per study group are needed. A loss to follow-up rate of 10% was also estimated. Therefore, the total target sample size was set at 208 participants ((2 × 98)/0.9 = 208). Participants who did not receive an ileostomy or colostomy during surgery were excluded and substituted with new inclusions. Data were analyzed according to the intention-to-treat protocol.

Statistical analyses of differences between the two groups were performed using IBM SPSS for Windows version 28.0. Baseline characteristics were summarized using descriptive statistics and compared between the intervention and control groups. Continuous data were reported as mean and standard deviation in case of normal distribution and as median and 95% confidence intervals in case of non-normal distribution. The normality of data distribution was analyzed by visually inspecting the histograms. Categorical variables were presented as frequencies and percentages. Independent t tests, Mann–Whitney *U* tests, Chi-squared tests, and Fisher’s exact tests were used to assess differences between groups as appropriate. Multivariate linear regression with stepwise backward selection was used to account for the potential confounding and stratifying factors. A two-tailed *p*-value ≤ 0.05 was considered statistically significant.

Patient history was categorized as follows: none, minimal, or extensive, with minimal history defined as one or two diseases generally not affecting or debilitating current quality of life (e.g., hypertension, appendectomy), and extensive history defined as having chronic diseases or several abdominal surgeries affecting or debilitating current quality of life.

Patient-Reported Outcome Measurements (PROMs) were included in the analysis if the patient completed at minimum 80% of the PROM-related questionnaires per domain. Missing data were corrected using the participants’ mean outcome of the (domain of the) PROM. For missing values, a cut-off value of 20% was applied.

## Results

**Fig. 2:**
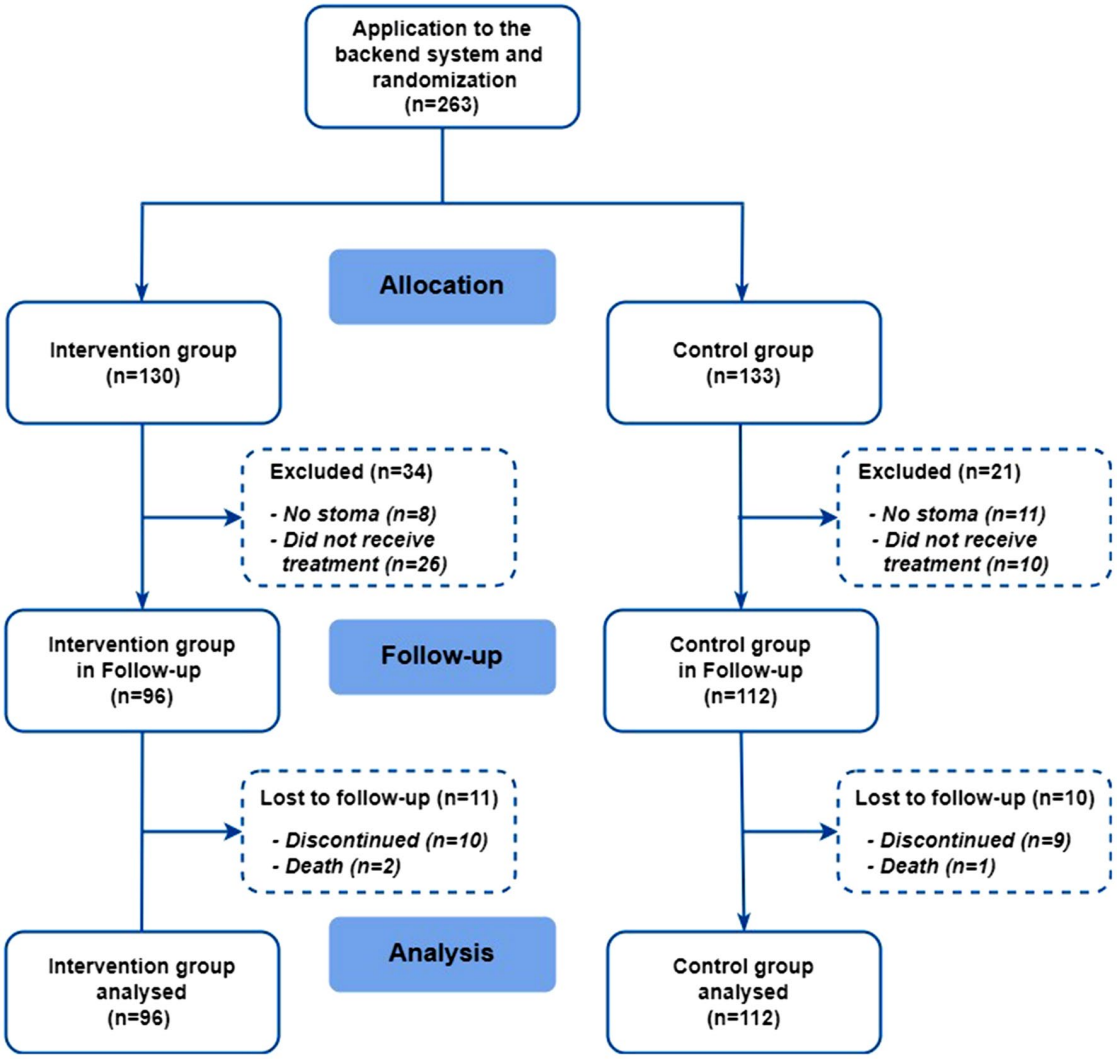
Treatment assignment and study flow

A total of 263 participants provided informed consent and were randomized. Of these participants, 36 participants did not receive the treatment allocation (did not download or use the Stoma App), 96 participants received the full version Stoma App (intervention group), and 112 received the restricted version of the Stoma App (control group, Fig. 2). The baseline characteristics of the participants, as presented in Table 1, were similar between the two groups except for a significantly worse overall preoperative performance score in the intervention group (87.0 vs 89.6, *p* = 0.041). The mean age of the study population was 56 years; the majority received a colostomy (59.6%) and the majority was operated upon in a non-acute, elective setting (63.5%). Both groups expressed overall sufficient scores on the mobile proficiency questionnaire. On average, patients in the elective setting started using the app 21 days before surgery, while patients in the emergency setting started using the app 5 days after surgery. From the patients in the intervention group, 20.1% utilized the peer contact function at least once.

**Table 1:** Baseline characteristics of study population

Variable			Intervention (*n* = 96)	Control (*n* = 112)	*p*-value
Male gender			54 (56.3%)	53 (47.3%)	0.213
Age			56.0 (13.4)	56.7 (14.8)	0.716
BMI			25.7 (4.5)	26.0 (4.7)	0.596
Karnofsky performance score			87.0 (9.2)	89.6 (9.4)	0.041
ASA					
1			7 (7.4%)	17 (16.0%)	0.265
2			67 (71.3%)	70 (66.0%)	
3			19 (20.2%)	17 (16.0%)	
4			1 (1.1%)	2 (1.9%)	
Patient history					
No patient history			23 (24.0%)	20 (17.9%)	0.377
Minimal patient history			26 (27.1%)	39 (34.8%)	
Extensive patient history			47 (49.0%)	53 (47.3%)	
Operation indication					
Benign			46 (47.9%)	59 (52.7%)	0.578
Malignant			50 (52.1%)	53 (47.3%)	
Operation setting					
Elective expected ostomy			57 (59.4%)	60 (53.6%)	0.506
Elective unexpected ostomy			5 (5.2%)	10 (8.9%)	
Emergency			34 (35.4%)	42 (37.5%)	
Type of ostomy					
Colostomy			53 (55.2%)	71 (63.4%)	0.258
Ileostomy			43 (44.8%)	41 (36.6%)	
Hospital setting					
Academic			26 (27.1%)	30 (26.8%)	1.000
Teaching			70 (72.9%)	82 (73.2%)	
Days to operation					
Elective			− 21.1 (42.8)	− 21.1 (43.8)	1.000
Emergency			4.8 (9.6)	2.7 (4.7)	0.227
Mobile proficiency^a^			68.4 (54.8–70.0)	65.4 (53.8–70.0)	0.219
General QoL					
Physical QoL			65.3 (20.6)	64.7 (24.0)	0.839
Psychological QoL			71.9 (14.7)	72.3 (14.0)	0.838
Social relationships^a^			83.3 (75.0–100)	83.3 (72.9–100)	0.404
Environment QoL			79.1 (13.5)	79.7 (14.7)	0.768
Disability score^a^			19.5 (15.0–27.5)	21.0 (15.0–30.3)	0.658

^a^Data presented as median (IQR)

The results on the Stoma QoL questionnaire at two weeks, one month, and three months postoperatively are presented in Table 2. At first sight, it appears that there were no significant improvements in the Stoma QoL for the intervention group. However, after adjusting for confounding factors using multivariate linear regression analysis, a significant improvement in reported quality of life was observed at the timestamp of one-month postoperative (Table 3). Confounders included the quality of life at baseline, the readmission rate, and reported psychological problems.

**Table 2:** The Stoma QoL of the intervention and control group

Stoma-QoL	Intervention	Control	*p*-value
2 weeks postoperative	55.5 (11.1)	53.9 (11.2)	0.357
1 month postoperative	56.3 (10.9)	54.9 (12.0)	0.416
3 months postoperative	58.4 (12.1)	56.9 (12.3)	0.401

The Stoma QoL is presented as mean with the standard deviation

**Table 3:** Multiple linear regression analysis of Stoma QoL

Variable		B	95.0% CI		Standard error	*p-*value	
			Lower	Upper			
Stoma QoL at 2 weeks							
(Constant)		30.738	21.894	39.583	4.479	< 0.001	
Intervention							
Intervention		1.929	− 1.116	4.973	1.542	0.213	
Control^a^		–	–	–	–		
Operation indication							
Benign		− 1.801	− 4.998	1.396	1.619	0.268	
Malign^a^		–	–	–	–		
Stoma							
Ileostomy		− 2.081	− 5.181	1.018	1.570	0.187	
Colostomy^a^		–	–	–	–		
Psychological QoL at baseline		0.361	0.251	0.472	0.056	< 0.001	
Medical history							
No history		− 0.532	− 4.659	3.595	2.090	0.799	
Minimal history		− 3.495	− 6.986	− 0.005	1.767	0.050	
Extensive history^a^		–	–	–	–	–	
Stoma QoL at 1 month							
(Constant)		39.182	28.8717	49.646	5.295	< 0.001	
Intervention							
Intervention		3.064	0.174	5.953	1.462	0.038	
Control^a^		–	–	–	–		
Operation indication							
Benign		0.989	− 2.112	4.089	1.569	0.530	
Malign^a^		–	–	–	–		
Stoma							
Ileostomy		1.123	− 1.858	4.103	1.508	0.458	
Colostomy^a^		–	–	–	–		
Psychological QoL at baseline		0.167	0.032	0.302	0.068	0.016	
Environment QoL at baseline		0.148	0.009	0.287	0.070	0.037	
Readmission within 1 month		− 6.628	− 10.955	− 2.301	2.189	0.003	
Self-reported psychological problems		− 11.791	− 15.250	− 8.333	1.750	< 0.001	
Stoma QoL at 3 months							
(Constant)		66.699	54.890	78.507	5.970	< 0.001	
Intervention							
Intervention		2.039	− 0.747	4.825	1.408	0.150	
Control^a^		–	–	–	–		
Operation indication							
Benign		1.870	− 1.289	5.030	1.597	0.244	
Malign^a^		–	–	–	–		
Stoma							
Ileostomy		0.568	− 2.337	3.472	1.468	0.150	
Colostomy^a^		–	–	–	–	–	
Psychological QoL at baseline		0.147	0.033	0.262	0.058	0.012	
Self-reported psychological problems		− 8.445	− 11.475	− 5.415	1.532	< 0.001	
Disability score at 3 months		− 0.762	− 1.013	− 0.512	0.127	< 0.001	
Operation setting							
Elective^a^		–	–	–	–	–	
Unexpected ostomy		− 0.780	− 6.138	4.578	2.709	0.774	
Emergency		− 3.440	− 6.622	− 0.257	1.609	0.034	

*B* beta coefficient for Stoma QoL, *CI* confidence interval

^a^Reference category

Patients in both groups had five contact moments (face to face or telephonic) at the outpatient clinical with a stoma nurse in the postoperative phase. Patients in academic medical centers had significantly fewer contacts in total, compared to patients in teaching hospitals (2.1 vs. 2.8 at 1 month p = 0.019; 1.5 vs 2.8 at 3 months *p* < 0.001). This was independent from the incidence of stoma-related problems, suggesting different postoperative pathways or low-threshold contact in teaching hospitals. Self-reported problems were present in both the intervention and control groups. Physical problems were reported by 74.3% vs. 69.4% of patients at 1-month (*p* = 0.500) and 68.5% vs. 65.6% at 3 month interval (*p *= 0.411). Similarly, psychological problems were reported by 72.2% vs. 73.2% of patients at 1 month (*p* = 1.000) and 68.1% vs. 64.8% at three months (*p* = 0.740). The readmission rate of the intervention group was significantly higher at 1 month after surgery (20.4% vs 10.0%, *p * = 0.047). Most readmissions were due to intra-abdominal abscesses (7.2%) or ileus (2.4%), see Table 4. The number of reported comorbidities in the intervention group was significantly lower (9.7% vs. 20.9%). Other clinical and patient-reported outcomes were comparable between the groups (Table 5).

**Table 4:** The indications for readmission indications

Indication for readmission	*N* = 208
Intra-abdominal abscess	14 (7.2%)
Ileus or no stoma output	5 (2.4%)
Nausea	2 (1.0%)
Revision stoma	2 (1.0%)
Dehydration and/or met electrolyte imbalance	2 (1.0%)
Pneumoniae	1 (0.5%)
Anastomotic leakage	1 (0.5%)
Wound infection	1 (0.5%)
Other	2 (1.0%)

**Table 5:** Secondary outcomes

Variable	Intervention	Control	*p*-value
Length of admission in days^a^	7.0 (5.0–11.5)	7.0 (5.0–11.3)	0.674
Ostomy related complications			
1 month	28 (32.2%)	34 (31.8%)	1.000
3 months	19 (23.2%)	24 (23.1%)	1.000
Other complications			
1 month	34 (38.6%)	34 (31.8%)	0.366
3 months	2 (2.4%)	4 (3.9%)	0.695
Comorbidities			
1 month	9 (9.7%)	23 (20.9%)	0.034
3 months	24 (28.2%)	29 (26.6%)	0.871
Readmissions			
1 month	19 (20.4%)	11 (10.0%)	0.047
3 months	18 (21.2%)	16 (14.7%)	0.258
Reoperations			
1 month	5 (5.4%)	13 (11.8%)	0.139
3 months	6 (7.1%)	9 (8.3%)	0.794
Outpatient contacts with stoma-nurse^a^			
1 month	2.5 (1.8)	2.7 (2.0)	0.296
3 months	2.6 (2.3)	2.4 (2.0)	0.211
Self-reported physical problems			
1 month	55 (74.3%)	68 (69.4%)	0.500
3 months	50 (68.5%)	61 (65.6%)	0.411
Self-reported psychosocial problems			
1 month	52 (72.2%)	71 (73.2%)	1.000
3 months	49 (68.1%)	59 (64.8%)	0.740
General QoL (at 1 months)			
Physical QoL	64.7 (16.8)	62.3 (20.5)	0.400
Psychological QoL	68.5 (14.5)	68.2 (14.3)	0.875
Social relationships^a^	83.3 (66.7–100)	83.3 (66.7–83.3)	0.292
Environment QoL	77.4 (13.1)	76.1 (15.4)	0.556
Psychosocial adjustment			
2 weeks	69.6 (7.9)	69.2 (7.3)	0.702
1 month	68.9 (7.5)	68.4 (7.3)	0.652
3 months	68.7 (8.6)	68.5 (8.6)	0.845
Disability assessment (3 months)^a^	17.0 (14.0–22.0)	17.5 (14.0–24.3)	0.812

^a^Data presented as median and interquartile range

Data presented as median (IQR)

## Discussion

Providing adequate stoma care is essential to help patients cope with their stoma and improve their QoL. To date, it is reported in literature that patients experience a lack of adequate and personalized information provision, postoperative care, and support and are in need of contact with peer patients especially when they are out of hospital [13, 14]. To address these shortcomings and optimize stoma care, a patient-centered mobile app tailored to meet the needs and preferences of stoma patients holds significant potential. This study examined the effects of having timely, individualized information, and peer contact available via the Stoma App on patients with ileostomies or colostomies, as well as the value of having information that is both accessible and trustworthy.

The intervention version of the Stoma App demonstrated a significant improvement in the stoma quality of life by 3.1 (*p* = 0.038) in the multivariate analysis, at one month after surgery. This finding holds significant importance, especially considering that the immediate postoperative period is often characterized by various insecurities and psychosocial challenges [21]. In this period, patients may not always have adequate self-efficacy, which may result in insecurity, social impairment, or isolation. In return, this may lead to an increase in emergency department visits without readmission (patients being insecure) [22], or in contrast, and even worse, to an increase in readmission (patients waiting too long to present themselves) [23]. In the longer postoperative period, patients generally have higher self-efficacy and thus may benefit less from the app. In our study, the Stoma App showed significant improvement in the primary outcome measurement ‘quality of life’ after correction for confounders, but not in the secondary outcome measures. Interestingly, the intervention group had a significantly higher readmission rate one month after surgery. This was primarily due to operation-related complications, such as intra-abdominal abscess or ileus (Table 4). It is highly unlikely that stoma-related guidance or peer contact have any influence on these complications, as the app does not provide any information, guidance, or advice related to these specific medical conditions, nor is provided information likely to be of influence. However, the significantly lower Karnofsky performance score of the intervention group may be attributed to the higher readmission rate. Co-morbidities were less frequently reported in the intervention group in the same period. This may result from underreporting in the intervention group, as complications or readmissions are likely to obscure other problems.

Two stoma-related apps have been described in literature with inconsistent user outcomes [24, 25]. These apps were less capacious in content and user interface than the Stoma App, lacking a proper (user-) design and development testing process. In contrast, development of the Stoma App was based on an assessment of the actual problems that patients themselves reported to encounter in stoma care and their specific needs and desired functionalities [13, 14]. To that end, we involved both patient associations and the stoma nurse association intensively [26–28]. Indeed, the target group and stakeholders were involved in the development of the app and in pilot testing, to ensure its usability and relevance. Possible features that the apps can offer to patients were explored in beta testing before the app was registered in the app stores. This is a vital step in building good apps, as apps can provide many ways of providing information.

Although apps have great potential to improve and support healthcare, it is crucial that these apps are thoughtfully designed in terms of content and user interface, maintain technical stability, and adhere to privacy and medical device legislation to ensure their effectiveness and safety [29]. When developing an app, one must realize that app features are sometimes costly to build, protect, and maintain; and there is a ‘nice to have’ and ‘need to have’ that needs to be explored. It is important to acknowledge that apps are at risk of poor implementation and underutilization in healthcare if not built well. Addressing these concerns is crucial, as apps have additional features and benefits in comparison to a website or digital paper, which may positively impact patient care [16]. Therefore, to optimize and prepare for future implementation in standard care, the Stoma App was provided by ostomy nurses to their patients in this trial. And also, our partner in development and spreading insights -the Dutch patient associations- propagated the app and patient stories about it on their website and in their newsletters.

We deliberately chose not to compare the full version of the app with ‘care as usual’ –as we expected this outcome evaluation would be biased. Normal routine of stoma care consists of a one-time informative conversation with a physician or stoma nurse before surgery, possibly supported by a paper folder or a referral to a website. Providing information on a stoma-especially if the conversation immediately follows a conversation in which the message is given that one is diagnosed with cancer or another illness, is often not remembered by patients [30]. Thus, it is highly likely that having easily accessible information in an app on the own smartphone as an extra to normal routine will be valued more highly than not having such an app. Therefore, we compared two versions of the app to strengthen the evidence supporting the app’s impact and adequately evaluate the effectiveness the design’s add-ons, as suggested by patients. As the app was built with a subsidiary that is to be depleted, insurers require robust evidence to financially support an app built as ‘software as a medical device.’ We aim to keep offering this app free of charge to all stoma patients, in and outside of the Netherlands for many more patients to benefit from. For that, one needs evidence on the effect of the app as a medical device in patients as a whole, while considering the proposed benefits of the costly elements.

Although there was a significant increase in the quality of life of patients using the intervention version of the Stoma App, the uptake and utilization of the app can be further optimized. It is important to acknowledge that the Stoma App suffered from technical issues during the trial. Some of these were not adequately addressed or resolved in a timely manner by the app developer. These technical issues mainly affected the timed information feature of sending out push notifications to patient. This must be considered a crucial component of the timed intervention version of the app. This issue has now been resolved in further development and scaling up the app, including migration of the app and choosing a different app developer. This needed to be done in order to futureproof and sustain the app implemented in normal clinical practice, fitting current and future technical and legal requirements, and operational stability. Building an app and researching it-even after committing to a pilot testing phase for technical issues- is a journey in itself. New insights are bound to be derived and are generated by actual use and implementation research itself. It is important to acknowledge this phenomenon, be transparent about it, and act accordingly. It is encouraging that despite the technical issues, on the primary outcome measurement significant difference was noted. This strengthens our belief that with optimal functionality, the value of personalization and peer support is likely to be higher than now visible.

The need for peer contact is frequently reported in literature by patients having a stoma [11, 13, 14, 22]. However, only one out of five patients in the intervention group used the peer contact function. This may indicate that, when asked, patients may have responded socially desirable to the question of whether peer support is important for them. It seems that for the majority of patients in our study, the opportunity to have peer contact via an app is not a ‘need to have’ feature, but rather a ‘nice to have.’ That said, one out of five patients used this feature, being either curious or in need of the support or opinion of a peer. It would be interesting to know, if these patients have a weaker social network than the ones who did not use it, but that could not be retrieved from data. And one may argue that one out of five is relevant number in itself to support the need for this feature.

Although the app is freely available in the app stores and publicized by patient associations, the involvement of local stoma nurses proved to be key in the process and success of the Stoma App [31]. The stoma nurses recruited and onboarded the patients for the trial, which took approximately 15 min. In addition, stoma nurses helped patients not familiar with app installation and with overcoming some digital literacy issues using the app. We consider this to be a best fit in the normal work routine, as patients in both groups needed a code to access the app. Of course, we needed to ensure that there were only patients having or getting a stoma as users in the app. Throughout the study, the participating stoma nurses were updated about course of the study and new app insights. Also, non-participating stoma nurses were informed about the trial and the app on national stoma congresses, many of them expressing interest in the app. In our study, as in many multicenter trials, patient recruitment varied between the study sites. That can be explained because some nurses actively integrated the app into the standard care pathway, while others did not and sometimes forget about the app.

This study has several limitations. Mostly importantly, it is highly likely that the results were significantly and negatively influenced by technical issues within the intervention version of the app. When developing a mobile app, careful consideration should be given to selecting a qualified app developer. But one should also clearly agree on what is included in app maintenance –and what are agreeable timeframes for maintenance—when an app is in trial. This, to ensure adequate support also after build and registration in app stores [15]. Although the developer possessed relevant certifications to ensure compliance to privacy and quality requirements, as well as having prior experience in the development of medical apps, the technical support and timely reaction time for this app proved to be inadequate. Especially for apps in medical trials, it is crucial to establish a solid agreement that obligates the developer to promptly detect and correct any technical problems that may arise. That said, even with the technical impairments now resolved, the intervention version of the app proved to be superior in supporting quality of life of stoma patients. Second, the intervention group had a significantly slightly worse preoperative clinical condition (and higher readmission rate) which may have negatively impacted the results. Third, the distribution of participants between the intervention and control groups was unequal, resulting from exclusions before receiving treatment (withdrawal, did not receive treatment), or because they did not receive an anticipated stoma. Consequently, this imbalance might have influenced the statistical significance of the results, as the differences in outcomes would need to be more substantial to be significant. Lastly, results may be biased as questionnaires were to be completed in the app itself. This method allowed participants to “click through” the questions quickly, potentially leading to less thoughtful answers and influencing the accuracy and reliability of the data collected.

To further explore and address the need for optimization of the uptake and utilization of the app, we are investigating facilitators and barriers in patients’ and stoma nurses’ engagement using semi-structured interviews. It is advised by our participating stoma nurses and authors incorporate the app into the care pathway, as the app requires limited time from personnel, it simulates consistent engagement and utilization by stoma nurses. By doing so, to provide more patients with the benefits of the app.

## Conclusion

The Stoma App—software as a medical device—improves the quality of life of stoma patients. This is a significant step forward in the optimization of stoma care. The app provides patients with ileostomies or colostomies with personalized support, peer contact if they need or desire to have such contact on a voluntary basis and a reliable, easily accessible base of information. This study demonstrated the app’s effectiveness in improving stoma quality of life in the critical postoperative period. Considering the study outcomes and the minimal time commitment required from healthcare personnel, it is highly recommended that the app be integrated into standard stoma care pathways.
